# Intrauterine human chorionic gonadotropin administration before embryo transfer (IHABT): an individual participant data meta-analysis of randomized controlled trials

**DOI:** 10.1093/humupd/dmag009

**Published:** 2026-04-16

**Authors:** Haowen Zou, Karim S Abdallah, Barbara Wirleitner, Kathleen H Hong, Isarin Thanaboonyawat, Pitak Laokirkkiat, Maryam Hafezi, Shoji Kokeguchi, Ahmed Makhlouf, Sol Libesman, David Nguyen, Jonathan G Williams, Marian Showell, Moustafa Gadalla, Ben W J Mol, Wentao Li, Rui Wang

**Affiliations:** Department of Obstetrics and Gynaecology, Monash University, Clayton, VIC, Australia; Department of Obstetrics and Gynaecology, Women’s Health Hospital, Faculty of Medicine, Assiut University, Assiut, Egypt; NextFertility IVF Centers Prof Zech Bregenz, Bregenz, Austria; IVIRMA New Jersey, Basking Ridge, NJ, USA; Infertility and Reproductive Biology Unit, Department of Obstetrics and Gynaecology, Faculty of Medicine Siriraj Hospital, Mahidol University, Bangkok, Thailand; Infertility and Reproductive Biology Unit, Department of Obstetrics and Gynaecology, Faculty of Medicine Siriraj Hospital, Mahidol University, Bangkok, Thailand; Department of Endocrinology and Female Infertility, Reproductive Biomedicine Research Centre, Royan Institute for Reproductive Biomedicine, Tehran, Iran; Hanabusa Women’s Clinic, Kobe, Japan; Department of Obstetrics and Gynaecology, Women’s Health Hospital, Faculty of Medicine, Assiut University, Assiut, Egypt; Faculty of Medicine, Badr University in Cairo, Cairo, Egypt; NHMRC Clinical Trials Centre, Faculty of Medicine and Health, University of Sydney, Sydney, NSW, Australia; NHMRC Clinical Trials Centre, Faculty of Medicine and Health, University of Sydney, Sydney, NSW, Australia; NHMRC Clinical Trials Centre, Faculty of Medicine and Health, University of Sydney, Sydney, NSW, Australia; Department of Obstetrics and Gynaecology, University of Auckland, Auckland, New Zealand; Department of Obstetrics and Gynaecology, Women’s Health Hospital, Faculty of Medicine, Assiut University, Assiut, Egypt; Department of Obstetrics and Gynaecology, Monash University, Clayton, VIC, Australia; Department of Obstetrics and Gynaecology, Amsterdam University Medical Centre, Amsterdam, The Netherlands; National Perinatal Epidemiology and Statistics Unit, Centre for Big Data Research in Health, The University of New South Wales, Sydney, NSW, Australia; NHMRC Clinical Trials Centre, Faculty of Medicine and Health, University of Sydney, Sydney, NSW, Australia

**Keywords:** intrauterine, human chorionic gonadotropin, embryo transfer, clinical pregnancy, live birth, individual participant data

## Abstract

**BACKGROUND:**

Intrauterine administration of hCG has been considered as a promising IVF add-on before embryo transfer to improve fertility outcomes. A Cochrane review and four more recent systematic reviews all showed improved clinical pregnancy rates and/or live birth rates following intrauterine administration of hCG, however, a high unexplained heterogeneity was also present.

**OBJECTIVE AND RATIONALE:**

To investigate the effectiveness and safety of intrauterine administration of hCG before embryo transfer in participants undergoing IVF. Individual participant data meta-analysis (IPD-MA) is recognized as the gold standard for evidence synthesis due to its ability to harmonize the data and to investigate treatment–covariate interactions. In addition, with recent experiences of guideline development and systematic review production raising increasing concerns about the trustworthiness of randomized controlled trials (RCTs) in women’s health research, an IPD-MA provides a unique opportunity to summarize the best available and most trustworthy evidence on this topic.

**SEARCH METHODS:**

We searched MEDLINE, Embase, Cochrane Gynaecology and Fertility Group Specialised Register, Cochrane Central Register of Controlled Trials, PsycINFO, and clinical trial registries without language restrictions up to January 2026. Inclusion criteria included RCTs comparing intrauterine administration of hCG before embryo transfer versus placebo or no intervention in participants undergoing IVF. The IPD Integrity tool and the TRACT checklist were used to evaluate the trustworthiness of studies with and without IPD, respectively. Both one-stage and two-stage random-effect IPD meta-analyses were performed with one-stage being the primary analysis.

**OUTCOMES:**

We detected 28 RCTs, of which 7 RCTs with IPD involving 2244 participants were included. All seven RCTs with IPD met trustworthiness criteria and six RCTs had overall low risk of bias. All RCTs without IPD did not meet trustworthiness criteria. IPD-MA showed intrauterine administration of hCG before embryo transfer did not improve live birth rates (7 RCTs, 2244 participants, odds ratio [OR] 0.99, 95% CI 0.83–1.19) or clinical pregnancy rates (7 RCTs, 2244 participants, OR 1.04, 95% CI 0.83–1.31). Studies without IPD showed different results from those with IPD for live birth (1.99, 0.72–5.50, *P* for interaction <0.001) and clinical pregnancy (1.87 (1.48–2.35), 17 RCTs without IPD, 3152 participants, *P* for interaction 0.005).

**WIDER IMPLICATIONS:**

Our IPD-MA has shown that intrauterine administration of hCG before embryo transfer is unlikely to improve the chance of clinical pregnancy and live birth. In the comparison between studies with IPD and without IPD, we found that none of the RCTs without IPD met trustworthiness criteria but showed a significant improvement in clinical pregnancy. We therefore suggest that intrauterine administration of hCG should not be offered as an IVF add-on in practice.

**REGISTRATION NUMBER:**

PROSPERO (CRD42020177397).

## Graphical Abstract

**Figure:**
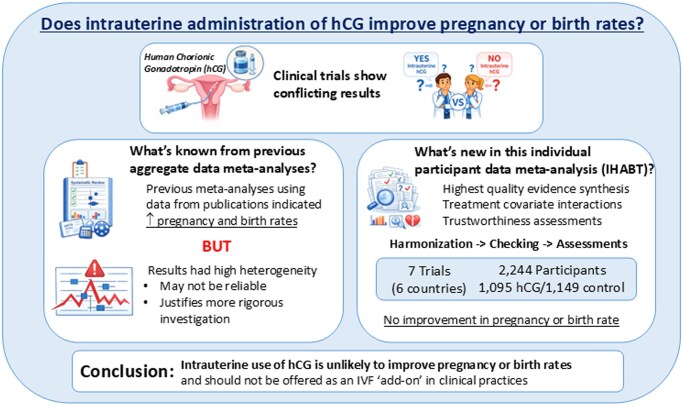
Graphical depiction of the analysis based on row-by-row data of studies examining the effect of intrauterine use of hCG on pregnancy and birth rates. IHABT, intrauterine HCG administration before embryo transfer.

## Introduction

Since the introduction of IVF in the late 1970s, its use has resulted in the birth of more than 12 million babies (Adamson, 2023). While the assisted reproductive techniques have been developed over the past two decades, the likelihood of live birth has remained largely similar, at around 30% per transfer cycle (Newman, 2023; Smeenk *et al.*, 2023). Therefore, in addition to the standard procedure of IVF treatment, medical supplements (Rubinstein *et al.*, 1999), alternative laboratory techniques (Kieslinger *et al.*, 2023), and other adjuvant therapies have been introduced and utilized in practices in attempting to improve outcomes. These subsidiary treatments are classified as ‘add-ons’ in IVF (Heneghan *et al.*, 2016). Despite IVF add-ons being frequently recommended and offered in practice (Spencer *et al.*, 2016), evidence and substantial investigation of the effectiveness and safety of most add-ons are lacking (Heneghan *et al.*, 2016; Harper *et al.*, 2017).

Among these proposed IVF add-ons, intrauterine administration of hCG has been considered a novel and promising approach (Tyler *et al.*, 2022). Intrauterine hCG is suggested to support trophoblast invasion, apposition, and adhesion to the endometrium, thus promoting the process of implantation (Kayisli *et al.*, 2003; Racicot *et al.*, 2014). Multiple randomized controlled trials (RCTs) have investigated the effect of intrauterine hCG on fertility outcomes and the findings were inconsistent. Subsequently, a Cochrane review on this topic showed improved clinical pregnancy rates after the administration of hCG (risk ratios [RR] 1.49 95% CI 1.32–1.68) (Craciunas *et al.*, 2018). Furthermore, four other more recent systematic reviews all suggested improvement of hCG in clinical pregnancy and/or live birth rates (Gao *et al.*, 2019; Tan *et al.*, 2019; Xie *et al.*, 2019; Tyler *et al.*, 2022). All these systematic reviews used the ‘all-inclusive approach’ of including all studies and none evaluated the impact of trustworthiness on results. Although evidence from meta-analyses on this topic presented the benefits of hCG in fertility outcomes, these meta-analyses also showed high unexplained heterogeneity across studies, making the findings difficult to interpret. The limited availability of subgroup data across trials and potential aggregation bias restricted the ability to perform subgroup analyses.

Individual participant data meta-analysis (IPD-MA) is recognized as the gold standard for evidence synthesis due to its ability to harmonize the data and to investigate treatment–covariate interactions (Riley *et al.*, 2020). In addition, with recent experiences of guideline development and systematic review production raising increasing concerns about the trustworthiness of RCTs in women’s health research (Mousa *et al.*, 2024; Wang, 2025), an IPD-MA provides a unique opportunity to summarize the best available and most trustworthy evidence on this topic. Therefore, we performed IPD-MA to evaluate the effectiveness and safety of intrauterine administration of hCG before embryo transfer in participants undergoing IVF (Tierney *et al.*, 2015).

## Methods

This IPD-MA was an international collaborative project, and the protocol was registered prospectively in the International Prospective Register of Systematic Reviews (PROSPERO No: CRD42020177397). It was reported following the Preferred Reporting Items for Systematic Reviews and Meta-Analyses (PRISMA) 2020 and the PRISMA-IPD extension (Stewart *et al.*, 2015; Page *et al.*, 2021). Ethical approval was obtained from the Monash University Human Research Ethics Committee (Project ID: 24096). Inform consent was not required for this IPD-MA as all participants in the original trials had provided informed consent for each trial and non-identifiable data was used for this IPD-MA.

### Literature search and eligibility criteria

MEDLINE, Embase, Cochrane Gynaecology and Fertility Group Specialised Register, Cochrane Central Register of Controlled Trials, and PsycINFO were searched to identify eligible studies. RCTs registry including clinicaltrails.gov and the World Health Organization International Clinical Trials Registry Platform (ICTRP) were searched. The search strategy was adopted from the latest Cochrane review on this topic (Craciunas *et al.*, 2018). The search was performed on 7 December 2022 with a top-up search performed on 27 January 2026 (Supplementary Table S1). There was no restriction in language. Both published and unpublished reports were eligible.

RCTs investigating the effectiveness and safety of intrauterine hCG administration before embryo transfer (IHABT) were included. Non-randomized and quasi-randomized studies were excluded. The participants were those with infertility undergoing embryo transfer. The intervention was intrauterine administration of hCG and the comparator was no intervention or a placebo.

### Screening

Two reviewers (H.Z., K.S.A., and M.G.) independently screened the titles and abstracts, followed by reviewing the full texts for eligibility. Conflicts were solved by a third reviewer (R.W.).

### Establishment of the IHABT (Intrauterine HCG Administration Before embryo Transfer) Collaboration

Corresponding authors of eligible studies were invited to share the de-identified individual participant-level data and join the IHABT Collaboration. We searched the published and unpublished reports, affiliation websites, and the latest reports, if any, of corresponding authors of included trials for up-to-date contact details. We sent the invitation emails to each corresponding author. When the contact details of the corresponding authors were not reachable, we attempted to contact other co-authors of the trials. We also contacted colleagues from the same country within our collaboration networks to assist us in reaching out to the corresponding authors who did not respond to the invitation emails. In all, we sent a follow-up email every 2 weeks and we attempted at least four rounds of emails to corresponding authors for those eligible trials who did not respond.

### Outcomes

The primary outcome was live birth, defined as the birth of a living baby after 20 weeks of gestation. Twin or multiple births in the same delivery were considered as a single live birth event. The secondary outcomes were clinical pregnancy, defined as pregnancies with positive cardiac pulsations confirmed by ultrasound at 4 weeks or more after embryo transfer; ongoing pregnancy, defined as pregnancy with confirmed foetal cardiac activity after complete 12 weeks of pregnancy; miscarriage, defined as spontaneous foetal loss after confirming the presence of foetal cardiac pulsations; multiple pregnancy, defined as a pregnancy with more than one foetus; ectopic pregnancy, defined as a pregnancy outside the uterine cavity, diagnosed by ultrasound, surgical visualization, or histopathology; and pelvic infection (endometritis, peritonitis, or pelvic abscess). Outcomes were pre-specified in the protocol and the protocol was sent to corresponding authors before data collection. After the IPD was received, outcomes were harmonized to maximize data availability.

### Data extraction and management

After the data access agreement was signed, data were transferred via ‘Monash Drive’, a data sharing and collaboration platform (ISO 27001 Certified) designed to transfer and store data. The data sharing approach was agreed upon by both the data provider and receiver, and no identifiable information was shared. In addition to the de-identified dataset, the data dictionary, protocol, ethical board approval and other documents related to the trial were also shared if available.

The IPD of each randomized participant was requested, regardless of whether they were included in the analyses of the original report or not. The received IPD were examined for internal consistency, validity, and missing data. To evaluate the reproducibility of included studies, baseline characteristics and the outcomes of each trial were tabulated and compared to those in the trial publications. The data was recoded and harmonized across all datasets.

### Trustworthiness assessment

We used the IPD Integrity Tool to assess the trustworthiness of included trials with IPD available (Hunter *et al.*, 2024). The tool comprises 31 items, including 13 study-level and 18 IPD-specific items. IPD-specific items involve eight domains, including unusual data patterns, baseline characteristics, correlations, data violations, pattern of allocation, internal and external inconsistencies, and plausibility of data.

For any discrepancy or identified issues, communications were made with the corresponding author of the specific trial, and a solution was obtained before we agreed to include the datasets for analyses. Two reviewers (S.L. and R.W.) assessed the trustworthiness of trials with IPD independently.

For studies without IPD, the checklist to assess Trustworthiness in RAndomised Controlled Trials (TRACT checklist; Mol *et al.*, 2023) was used to evaluate trustworthiness criteria when the full-text publication was available. The TRACT checklist included the following seven domains comprising 19 items on governance, author group, plausibility of intervention usage, timeframe, drop-out rates, baseline characteristics, and outcomes (Mol *et al.*, 2023). A study was rated major concerns if at least one domain was rated as major concerns. One reviewer (D.N. or J.G.W.) evaluated the trustworthiness of trials without IPD, with discussions with R.W. to reach consensus.

### Risk of bias assessment

We used the Cochrane risk of bias 2.0 tool to assess the following domains: bias arising from the randomization process, bias due to deviations from intended interventions, bias due to missing outcome data, bias in measurement of the outcome, and bias in selection of the reported result (Sterne *et al.*, 2019). Two reviewers (H.Z., K.S.A., and M.G.) performed the risk of bias assessments independently. A third reviewer (R.W.) was involved in solving any disagreement.

### Overall certainty of evidence

The GRADE (Grading of Recommendations Assessment, Development, and Evaluation) approach was used to assess the overall certainty of evidence (Guyatt *et al.*, 2011).

### Statistical analysis

Intention-to-treat principle was used as the main analysis for all outcomes. Both one-stage and two-stage random-effect IPD meta-analyses were performed with one-stage being the primary analysis. In the one-stage approach, we accounted for the clustering effect of the participants within each study in a logistic regression by using a stratified intercept by study, random treatment effect, and maximum likelihood estimator (Thomas *et al.*, 2014; Riley *et al.*, 2021). In the two-stage method, we used restricted maximum likelihood estimator with Hartung–Knapp–Sidik–Jonkman variance correction (Burke *et al.*, 2017; Riley *et al.*, 2021). *I*^2^ statistic was used to measure percentage of total variability due to heterogeneity and tau^2^ statistic was also reported. As all outcomes of interest in our study are dichotomous outcomes, we calculated the odds ratios (ORs) with 95% CIs. The unit of analysis was per woman randomized for each outcome.

Subgroup analyses were performed to estimate the treatment–covariate interactions on both live birth and clinical pregnancy whenever applicable, including type of comparator (placebo vs no intervention), previous implantation history (yes vs no), dosage of hCG (500 IU vs ≥500 IU), type of hCG (urinary hCG vs recombinant), injection timing (on day of ET vs ≥2 days before ET), transfer method (fresh vs frozen), and embryo transfer stage (cleavage vs blastocyst). When applicable, participant-level subgroup analysis was performed by synthesizing within-study interaction in a two-stage approach to avoid aggregation bias (Riley *et al.*, 2020; Godolphin *et al.*, 2023). Otherwise, for study-level subgroups, findings were presented in different strata of the subgroups.

To evaluate the differences between evidence from studies with and without IPD, we compared the summary ORs and 95% CIs. Contour-enhanced funnel plot was used to illustrate small-study effects.

All analyses were performed in Stata/SE (Standard edition) 18.0 (StataCorp LLC, TX, USA).

### Patient and public involvement statement

Patient or public members were not involved in this IPD-MA regarding initiating research project, defining research questions, or performing analysis. One patient and one public representative contributed to the interpretation of the findings, manuscript revision and they will also be involved in dissemination.

## Results

### Search results

**Figure 1.**
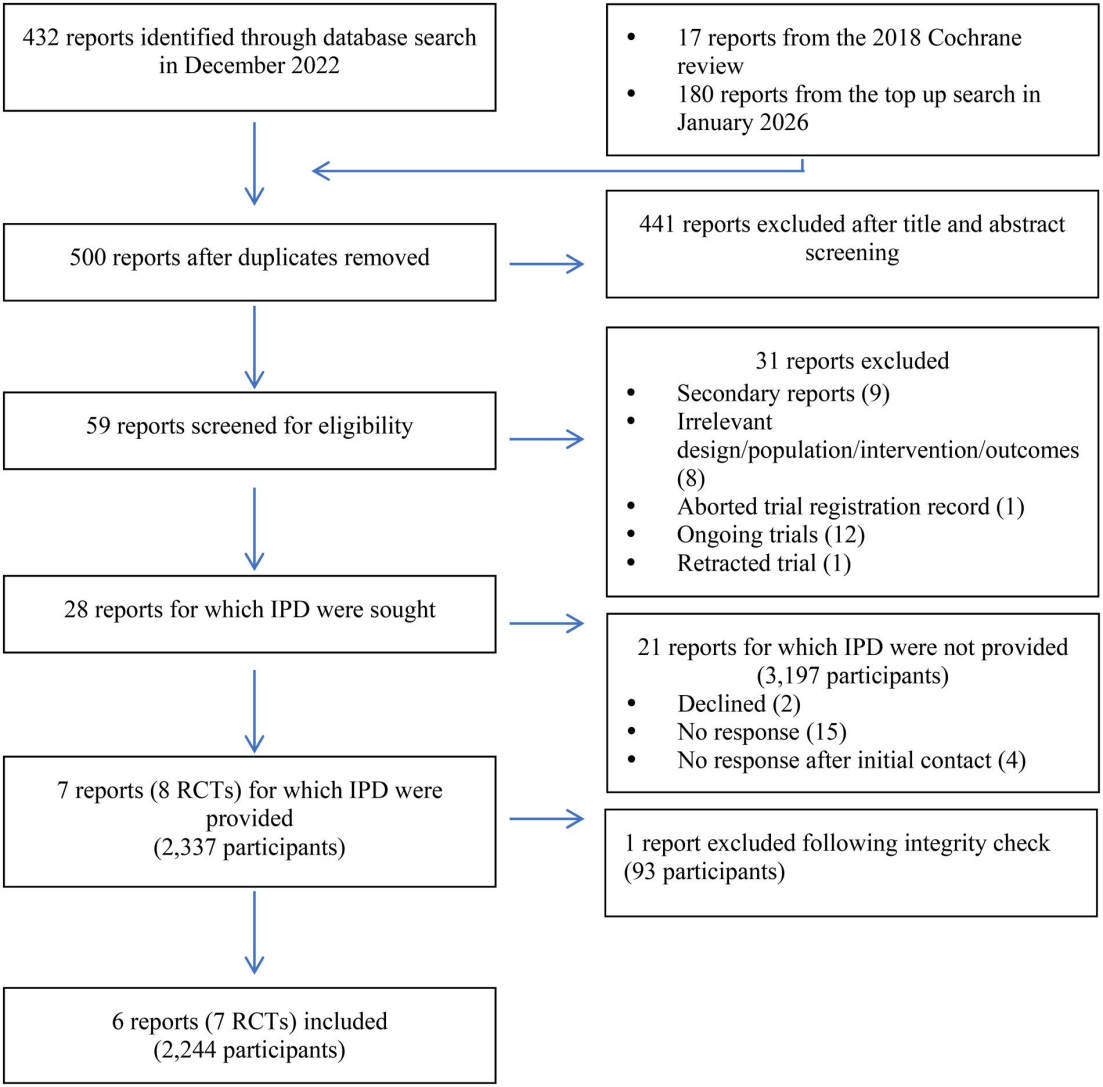
**Preferred reporting items for systematic review and meta-analysis of individual participant data (PRISMA IPD) flow diagram.** RCT, randomized controlled trial.

We identified 28 eligible reports on 5534 participants after the systematic search. Of these reports, 7 comprising 8 RCTs involving 2337 participants shared their IPD. This represents 42% of all eligible participants. Among the 21 reports from which we could not obtain IPD, authors did not response at all (N = 15) or did not respond after the initial contact (N = 4). One could not share data due to the decease of the corresponding author and one due to regulatory reasons (Fig. 1). A detailed list of eligible studies not sharing IPD and reasons for not participating is listed in Supplementary Table S2. The characteristics of excluded reports and ongoing registered trials are presented in Supplementary Table S3.

### Trustworthiness assessment

For the eight trials with IPD, the trustworthiness assessment was performed using the IPD Integrity tool and the results are presented in Supplementary Table S4. Aggregate data/publication-level check was performed in all eight trials (Supplementary Table S4). One trial had major concerns due to a history of multiple retractions related to research integrity, and was therefore excluded from the analysis. The remaining seven trials had no major concerns.

For the 21 reports without IPD, 14 had full text available and 7 were abstract-only reports. The trustworthiness assessment was performed using the TRACT checklist for the 14 full-text reports. All 14 trials did not meet the trustworthiness criteria because of major concerns in at least one domain of the TRACT checklist, especially issues regarding trial registration (no or retrospective registration), sample size and/or the primary outcome. The details of the TRACT checklist findings are presented in Supplementary Table S5.

### Characteristics of included studies

The seven included RCTs comprised 2244 participants, equivalent to 41% of the overall participants reported to be randomized (Hong *et al.*, 2014; Matsumoto and Kokeguchi, 2015; Wirleitner *et al.*, 2015; Hafezi *et al.*, 2018; Laokirkkiat *et al.*, 2019; Abdallah *et al.*, 2021). All seven studies used urinary hCG in the intervention group. Six studies were placebo-controlled. Six studies used 500 IU hCG and one study used 1000 IU hCG (Table 1).

**Table 1.** **Characteristics and demographic information of included trials**.

First author, year	Country	Journal/conference	Study population	Fresh/frozen transfer	# ET	ET stage	Control group	HCG type	HCG timing	HCG dose	Outcomes of interest	N
Abdallah (2021)	Egypt	Reprod Biomed Online	Unselected	Fresh/frozen	1–3	Cleavage/blastocyst	Culture medium (0.1 ml)	Urinary	4 mins before ET	500 IU	Live birth, ongoing pregnancy, clinical pregnancy, miscarriage, ectopic pregnancy, multiple gestations	181
Hafezi (2018)	Iran	Archives of Gynecology and Obstetrics	Failed fresh transfer cycle	Frozen	1–4	Cleavage	Culture medium (40 μl) or no treatment	Urinary	7–10 mins before ET	500 IU	Live birth, clinical pregnancy, miscarriage, ectopic pregnancy, multiple gestations	180
Hong (2014)	USA	Fertility and sterility	Unselected	Fresh/frozen	1–2	Blasto-cyst	Culture medium (20 μl)	Urinary	≤3 min before ET	500 IU	Live birth, ongoing pregnancy, clinical pregnancy, miscarriage, multiple pregnancy, ectopic pregnancy	300
Matsumoto (2015)	Japan	ASRM abstract	Unselected	Frozen	1	Blasto-cyst	Culture medium (100 μl)	Urinary	3 days before ET	1000 IU	Live birth, clinical pregnancy, miscarriage, ectopic pregnancy	197
Laokirkkiat (2019)	Thailand	Archives of Gynecology and Obstetrics	Unselected	Fresh/frozen	1–3	Cleavage/blastocyst	Culture medium (10 μl)	Urinary	4 min before ET	500 IU	Live birth, clinical pregnancy, multiple gestation, miscarriage	200
Wirleitner (2015)a*	Austria	Reproductive Biology and Endocrinology	Failed fresh transfer cycle	Fresh	1–2	Blasto-cyst	Culture medium (40 μl)	Urinary	2 days before ET	500 IU	Live birth, clinical pregnancy, multiple gestation, miscarriage, ectopic pregnancy	182
Wirleitner (2015)b*	Austria	Reproductive Biology and Endocrinology	Unselected	Fresh	1–2	Blasto-cyst	Culture medium (40 μl)	Urinary	3 min before ET	500 IU	Live birth, clinical pregnancy, multiple gestation, miscarriage, ectopic pregnancy	1004

# ET, number of embryo transferred; ET, embryo transfer; ASRM: American Society for Reproductive Medicine.

* The two trials were reported in the same publication.

Of 2244 participants, 1095 had been allocated to the hCG group and 1149 to the control group (Table 2). The mean age was 35.9 years in the hCG group and 35.4 years in the control group. Seventy-one percent of participants in the hCG group and 67% in the control underwent a fresh embryo transfer cycle, with 84% and 79% having blastocyst transfers in the hCG group and the control group, respectively. Over 55% of participants had at least 2 embryos transferred.

**Table 2.** Characteristics of participants and interventions.

		HCG (n = 1095)	Control (n = 1149)
**Female age (mean, SD)** *		35.9 (4.5)	35.4 (4.7)
**BMI (mean, SD)** ^#^		23.5 (4.4)	23.7 (4.5)
**Infertility type** ^^^			
	Primary	495 (58.3%)	534 (59.5%)
	Secondary	354 (41.7%)	364 (40.5%)
**Infertility factor** ^$^			
	Female	143 (19.1%)	151 (18.9%)
	Male	324 (43.3%)	342 (42.9%)
	Mixed	155 (20.7%)	181 (22.7%)
	Unexplained	126 (16.8%)	124 (15.5%)
**Transfer method**			
	Fresh	781 (71.3%)	766 (66.7%)
	Frozen	314 (28.7%)	383 (33.3%)
**Number of embryos transferred**			
	1	376 (34.3%)	381 (33.2%)
	2	629 (57.5%)	654 (56.9%)
	3	90 (8.2%)	109 (9.5%)
	4	0	5 (0.4%)
**Stage of transfer**			
	Cleavage	179 (16.3%)	241 (21.0%)
	Blastocyst	916 (83.7%)	908 (79.0%)

* 1 missing in the control group.

# 315 missing in the hCG group, 317 missing in the control group.

^ 246 missing in the hCG group, 251 missing in the control group.

$ 347 missing in the hCG group, 351 missing in the control group.

### Risk of bias assessment

**Figure 2.**
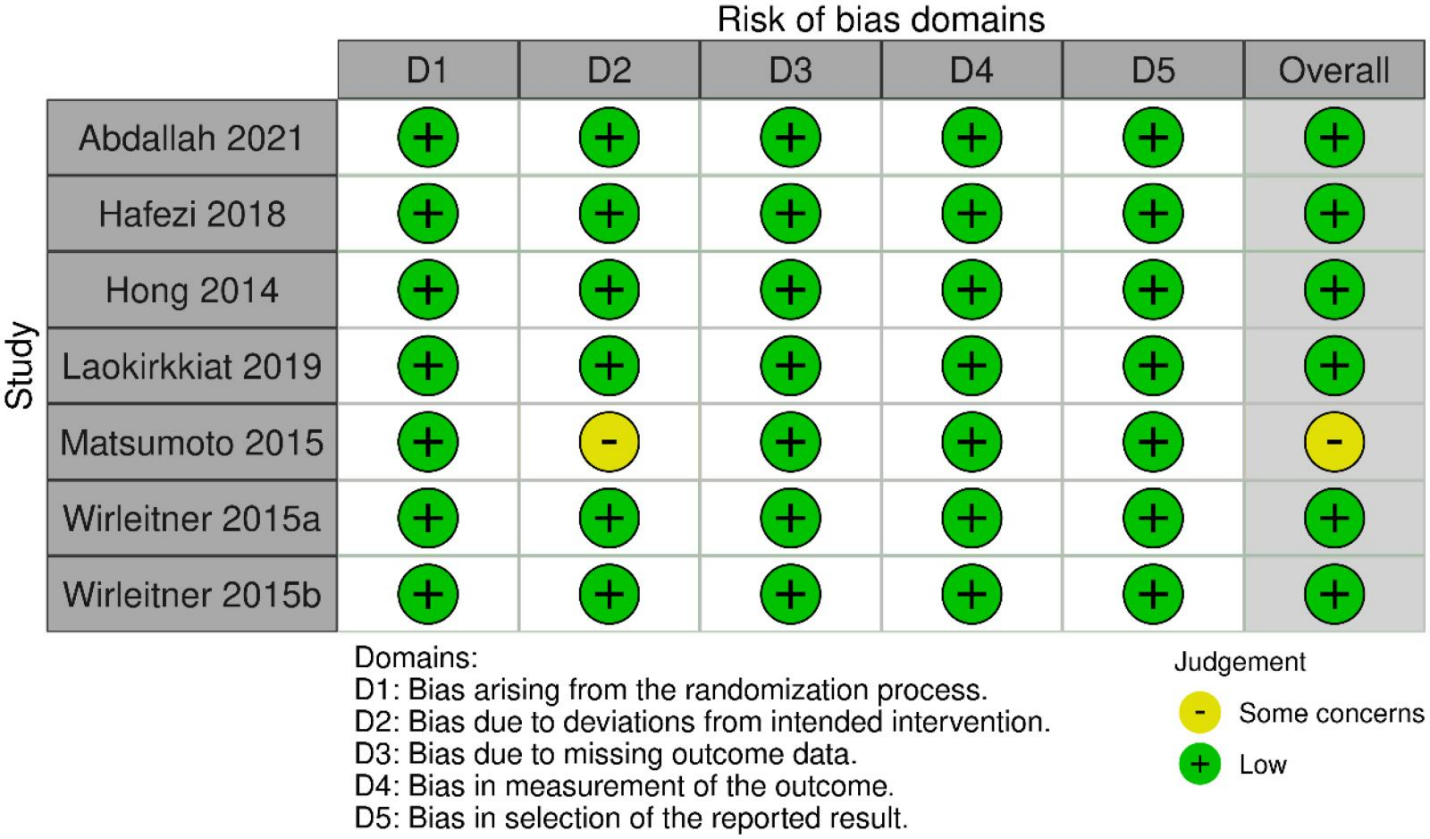
**Risk of bias assessment results using the Cochrane Risk of Bias 2.0 Tool.** All trials were assessed as low risk of bias for all domains, except for Matsumoto *et al*. with ‘some concerns’ in bias due to deviations from the intended interventions.

The risk of bias assessment was performed for the seven trials (Fig. 2). Six trials were assessed as ‘low risk’ of bias for all domains, and one trial was assessed as ‘some concerns’ in bias due to deviations from the intended interventions (Fig. 2).

### Primary outcome—live birth

**Figure 3.**
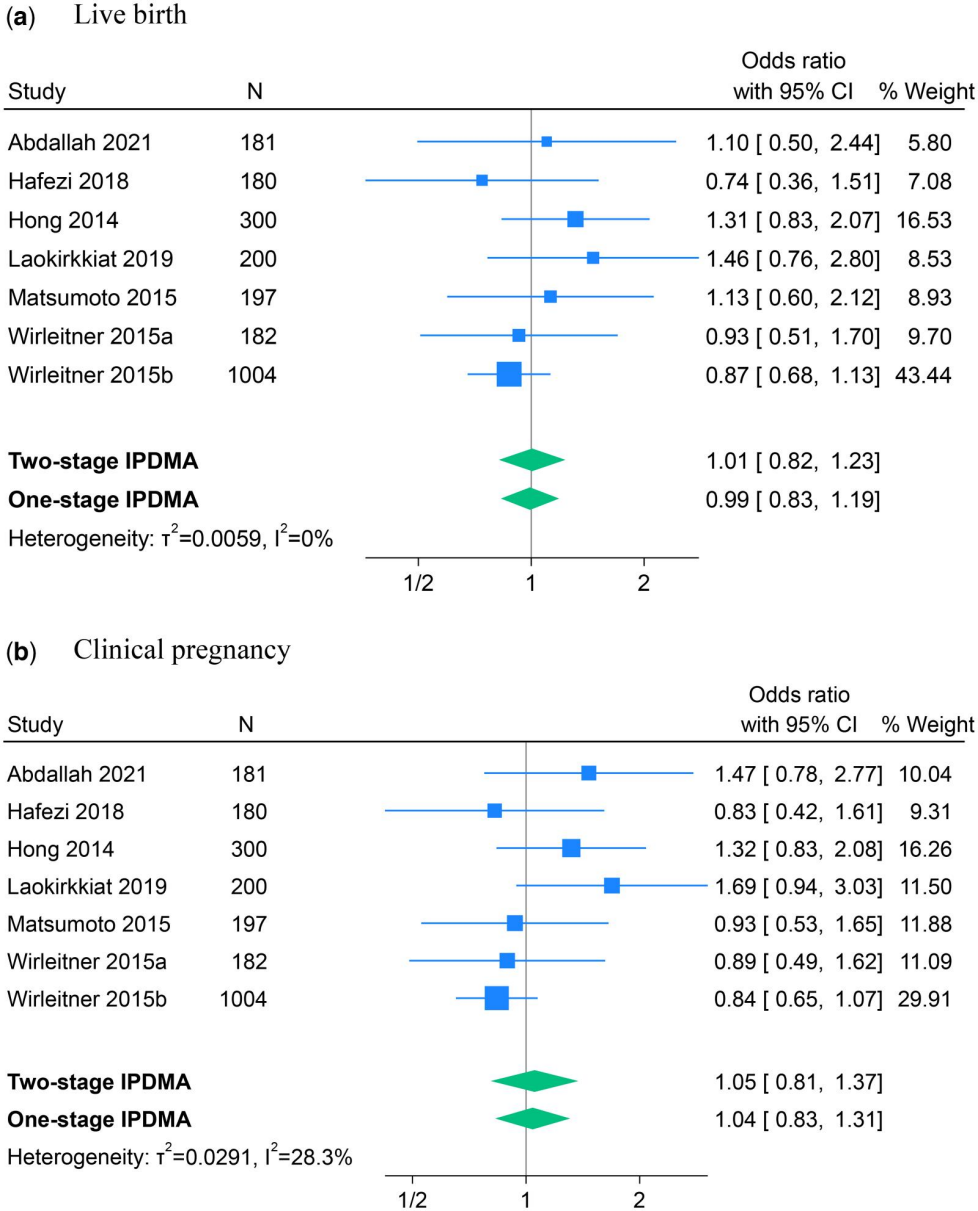
**Forest plots for live birth and clinical pregnancy.** The squares and lines represent the odds ratios and 95% CIs for each study and the diamonds refer to the summary odds ratios and 95% CIs from two-stage and one-stage IPDMA, respectively. (**a**) Live birth, (**b**) Clinical pregnancy IPDMA, individual participant data meta-analysis.

All seven trials included in the study reported the primary outcome (live birth). Intrauterine administration of hCG before embryo transfer does not improve live birth rates (one-stage OR: 0.99 (0.83–1.19); two-stage OR: 1.01 (0.82–1.23), *I*^2^ = 0%, Tau^2^ = 0.0059, moderate certainty of evidence, Fig. 3a).

### Secondary outcomes

The results of the secondary outcomes are presented in Table 3. Seven trials reported clinical pregnancy. The one-stage model showed an OR of 1.04 (0.83–1.31) with the two-stage model showing an OR of 1.05 (0.81–1.37, *I*^2^ = 28.3%, Tau^2^ = 0.0291). The forest plot is presented in Fig. 3b.

**Table 3.** One-stage meta-analysis for all outcomes.

Outcomes	Number of RCTs	Number of participants	Odds ratio	95% CIs	Overall certainty of evidence
**Live birth**	7	2244	0.99	0.83–1.19	Moderate*
**Clinical pregnancy**	7	2244	1.04	0.83–1.31	Moderate*
**Ongoing pregnancy** ^#^	7	2244	1.01	0.84–1.20	Moderate*
**Multiple pregnancy**	7	2244	1.44	0.76–2.76	Low**
**Miscarriage**	7	2244	1.21	0.89–1.63	Low**
**Ectopic pregnancy**	4	858	0.44	0.11–1.71	Low**

* Downgraded for one level due to imprecision.

** Downgraded for two levels due to imprecision.

# Live birth was used as a surrogate for ongoing pregnancy for Hafezi (2018), Laokirkkiat (2019), and Wirleitner (2015) (two trials reported in the same publication).

RCTs, randomized controlled trials.

The ORs for ongoing pregnancy, multiple pregnancy, miscarriage, and ectopic pregnancy based on the one to stage model were 1.01 (0.84–1.20), 1.44 (0.76–2.76), 1.21 (0.89–1.63), and 0.44 (0.11–1.71), respectively. The overall certainty of evidence was graded moderate for clinical and ongoing pregnancy and low for multiple pregnancy, miscarriage, and ectopic pregnancy, due to imprecision (Table 3). Analyses based on the two-stage model were overall consistent with the findings of the one-stage model for all outcomes (Supplementary Table S6).

### Treatment–covariate interaction

The individual participant-level treatment–covariate interaction analyses were performed for the following subgroups: type of transfer (frozen vs fresh) and embryo stage at transfer (blastocyst vs cleavage). There was no evidence of treatment-covariate for either live birth or clinical pregnancy (Supplementary Table S7).

The study-level subgroup analyses were performed for the following subgroups: type of control (placebo and no treatment), dosage of hCG (500 IU and 1000 IU), type of transfer (frozen and fresh), and embryo stage at transfer (blastocyst stage and cleavage stage). Similarly, no evidence of subgroup differences was found (Supplementary Table S8).

### Comparison between studies with and without IPD

**Figure 4.**
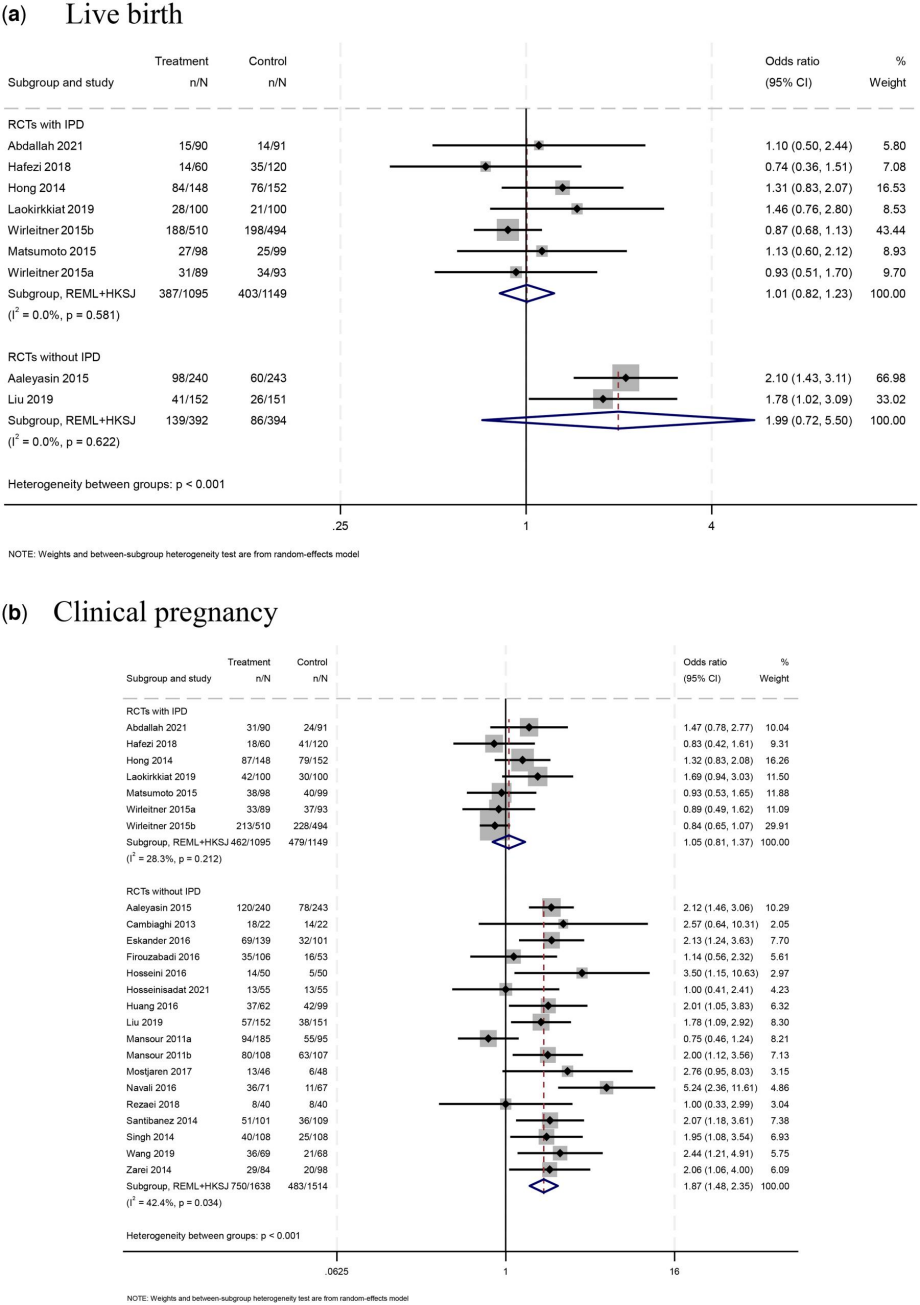
**Forest plots for live birth and clinical pregnancy, stratified by RCTs with and without IPD.** The forest plot was stratified by RCTs with and without IPD. The squares and lines represent the odds ratios and 95% CIs for each study and the diamonds refer to the summary odds ratios and 95% CIs from RCTs with and without IPD, respectively. (**a**) Live birth, (**b**) Clinical pregnancy. RCT, randomized controlled trials; IPD, individual participant data; REML, restricted maximum likelihood method; HKSJ, Hartung–Knapp–Sidik–Jonkman method.

The forest plots for live birth and clinical pregnancy, stratified by trials with and without IPD are presented in Fig. 4a and b. The pooled OR of live birth and clinical pregnancy for trials without IPD was 1.99 (0.72–5.50, 2 RCTs, 786 participants) and 1.87 (1.48–2.35, 17 RCTs, 3152 participants), respectively (Fig. 4a and b). These were significantly different pooled ORs based on RCT with IPD (interaction *P* values: <0.001 for live birth and 0.005 for clinical pregnancy). Smaller trials without IPD were more likely to show statistically significant findings, with asymmetrical distribution of the funnel plot for clinical pregnancy (Supplementary Fig. S1a and b). This indicates that the differences between evidence from studies with and without IPD could be mainly driven by large effects among small trials with trustworthiness concerns that did not provide IPD.

## Discussion

### Summary of key findings

This IPD-MA showed that intrauterine hCG before embryo transfers is unlikely to improve the chances of live birth or clinical pregnancy, although small effects could not be ruled out. There is no evidence in the subgroup analyses that embryo stage at transfer, type of transfer, or previous implantation failures modify the effect of intrauterine administration of hCG. None of the RCTs without IPD met trustworthiness criteria and their pooled analysis showed a large, favourable effect of hCG.

### Strengths and limitations

The strengths of this IPD-MA include standardization of the population, outcomes and analysis, retrieval of grey literature, trustworthiness assessment of included trials, and retrieval of additional participant-level information that was not available in the published reports. These enabled us to examine the true treatment effect of intrauterine administration of hCG before embryo transfer in participants undergoing IVF.

There are also some limitations. First, we were not able to collect all available IPD. Nevertheless, we collected IPD from the largest, highest-quality, and most trustworthy trials on this topic. Second, due to the limited availability of data on some baseline characteristics, we were restricted in performing some subgroup analyses. Thirdly, we requested outcomes with specific definitions (e.g. live birth after 22 weeks of gestation) before the transfer of data, but some of the IPD we received still varied in their definitions (e.g. live birth varied from 20 to 28 weeks of gestation). Lastly, we acknowledge that the TRACT checklist was developed by expert opinion followed by a pilot phase of testing, yet it has not been formally validated. Judgement of some domains may be considered subjective. As the TRACT checklist combines multiple domains, in our case did not change the overall findings on trustworthy assessments for each included study.

### Clinical and research implications

The ESHRE add-ons working group published Good Practice Recommendations on IVF add-ons in 2023 (Lundin *et al.*, 2023). They cited a Cochrane review (Craciunas *et al.*, 2018) and four other meta-analyses on this topic (Hou *et al.*, 2018; Gao *et al.*, 2019; Tan *et al.*, 2019; Xie *et al.*, 2019), all of which showed a potential benefit of intrauterine use of hCG, largely based on overlapping sets of included studies. Although the ESHRE add-ons working group did not recommend the use of hCG due to conflicting findings in efficacy and uncertainties on safety concerns, hCG is still prescribed as a regular IVF add-on in some clinics (Lensen *et al.*, 2021), partly due to the strong treatment effects in improving fertility outcomes reported in these systematic reviews. However, based on the best available and trustworthy evidence from this study, this practice is questionable.

The Cochrane review identified clear subgroup effects based on dose of hCG and stage of embryo transfer (Craciunas *et al.*, 2018), which are biological plausible. However, these need to be interpreted with caution, as the differences are only based on between-study differences instead of within-study differences. In other words, the observed differences could be due to other differences between studies in the subgroup analysis instead of the subgroup effect investigated in the Cochrane review. For studies included in our IPDMA, six used 500 IU hCG and one used 1000 IU hCG, which may all be considered above the threshold to manifest the treatment effect. In our IPDMA, it has the potential to more precisely investigate the subgroup effect based on within-study differences only, but we must acknowledge that we are underpowered here due to small number of studies reporting both blastocyst and cleavage stage transfer. Four studies had blastocyst transfer, two had a mixture of cleavage/blastocyst transfer, one had cleavage-only transfer. Blastocyst transfer account for ∼80% of included cycles. From a biological point of view, the effect of intrauterine administration of hCG may be less effect in blastocyst transfer cycles due to shorter exposure before implementation and therefore our finding is less representative for cleavage stage cycles due to the small proportion of these cycles included.

Our IPD-MA clearly showed unlikely benefit of intrauterine hCG in improving live birth and clinical pregnancy, refuting previous claims about its benefit on fertility outcomes. There has been increasing concerns about the trustworthiness of trials in women’s health research which may mislead consumers and clinical practitioners. For instance, the recent international guideline on polycystic ovary syndrome excluded >40% of eligible trials following integrity assessments (Mousa *et al.*, 2024).

Several other IPD-MAs in women’s health have reported patterns where trials with IPD showed no or only weak treatment effect, while trials without IPD showed large, favourable effects for the treatment of interest (Wang *et al.*, 2019; EPPPIC, 2021; Wessel *et al.*, 2022). In extreme cases, no studies were left for evidence synthesis after integrity checks (Wang, 2025). In this IPD-MA, the difference between evidence from RCTs with and without IPD was likely attributable to widespread major concerns about the trustworthiness of RCTs that did not provide IPD. This is in line with earlier evidence indicating that IPD sharing is a good indicator for better quality and more trustworthy research (Bordewijk *et al.*, 2020; Patabendige *et al.*, 2025). Therefore, in clinical guideline development to inform practice, IPD-MAs should be prioritized over aggregate meta-analysis. Moreover, the trustworthiness and quality of the original research should always be assessed before evidence synthesis, irrespective of whether IPD were shared.

With increasing data sharing requirements from funding bodies, journals, and publishers, future trials should adopt better data sharing policies to facilitate reproducibility and enable high-quality secondary research—maximizing the value of trial participants’ contributions and advancing the broader scientific community. Additionally, future trials investigating hCG administration before IVF should report on the full range of outcomes outlined in the core outcome set for infertility research (Duffy *et al.*, 2020), as well as key obstetrical and perinatal safety outcomes such as preeclampsia and hypertensive disorders, to better inform decision-making. As mentioned earlier, patients with a history of implantation failure (including repeated implementation failure) and those undergoing cleavage transfer were underrepresented in trials included in this IPD-MA, although they might benefit from intrauterine hCG administration from a biological point of view. Therefore, they merit further investigation in future trials. Finally, single embryo transfer becomes the standard practice in modern IVF and should be incorporated in the design of future trials.

## Conclusion

Intrauterine administration of hCG before embryo transfer is unlikely to improve live birth rate or other fertility outcomes in individuals undergoing IVF, with low-moderate evidence available. We did not identify any patient subgroup that would benefit from the intervention. The differences between evidence from RCTs with and without IPD were likely due to major concerns about the trustworthiness of RCTs without IPD. Therefore, intrauterine hCG before embryo transfer should not be offered as an IVF add-on in clinical practice.

## Supplementary Material

dmag009_Supplementary_Data

## Data Availability

The data custodians of the data of included trials retain the ownership of the trial data. Request for access to the IPD should be directed to individual trialists. Data dictionary and code for analysis will be made available upon reasonable request.
