# Supplementary material for: Intrauterine human chorionic gonadotropin administration before embryo transfer (IHABT): an individual participant data meta-analysis of randomized controlled trials
Source: Hum Reprod Update. 2026 Apr 16;32(4):458–70. doi: 10.1093/humupd/dmag009 (PMC13319334; doi:10.1093/humupd/dmag009)
Supplement: dmag009_Supplementary_Data [file dmag009_supplementary_data.docx]

**Intrauterine** **human chorionic gonadotropin administration before embryo transfer (IHABT): an individual participant data meta-analysis of randomised controlled trials - Supplementary tables and figures**

Haowen Zou^1^, Karim S. Abdallah^2^, Barbara Wirleitner^3^, Kathleen H. Hong^4^, Isarin Thanaboonyawat^5^, Pitak Laokirkkiat^5^, Maryam Hafezi^6^, Shoji Kokeguchi^7^, Ahmed Makhlouf^2,8^, Sol Libesman^9^, David Nguyen^9^, Jonathan G. Williams^9^, Marian Showell^10^, Moustafa Gadalla^2^, Ben W.J. Mol^1,11^, Wentao Li^12*^, Rui Wang^9*^

1. Department of Obstetrics and Gynaecology, Monash University, Clayton, VIC, Australia
2. Department of Obstetrics and Gynaecology, Women’s Health Hospital, Faculty of Medicine, Assiut University, Assiut, Egypt
3. NextFertility IVF Centers Prof Zech Bregenz, Bregenz, Austria
4. IVIRMA New Jersey, Basking Ridge, New Jersey, USA
5. Infertility and Reproductive Biology Unit, Department of Obstetrics and Gynaecology, Faculty of Medicine Siriraj Hospital, Mahidol University, Bangkok, Thailand
6. Department of Endocrinology and Female Infertility, Reproductive Biomedicine Research Centre, Royan Institute for Reproductive Biomedicine, Tehran, Iran
7. Hanabusa Women’s Clinic, Kobe, Japan
8. Faculty of Medicine, Badr University in Cairo, Cairo, Egypt
9. NHMRC Clinical Trials Centre, Faculty of Medicine and Health, University of Sydney, Sydney, NSW, Australia
10. Department of Obstetrics and Gynaecology, University of Auckland, Auckland, New Zealand
11. Department of Obstetrics and Gynaecology, Amsterdam University Medical Centre, Amsterdam, The Netherlands
12. National Perinatal Epidemiology and Statistics Unit, Centre for Big Data Research in Health and School of Women’s and Children’s Health, The University of New South Wales, Sydney, NSW, Australia

*Joint senior authors

Table of Contents

[Supplementary Table S1. Search strategies 3](#_Toc203638418)

[Supplementary Table S2. Characteristics of non-participated reports 11](#_Toc203638419)

[Supplementary Table S3. Characteristics of excluded reports and ongoing trials 15](#_Toc203638420)

[Supplementary Table S4. Trustworthiness assessment for trials with IPD 19](#_Toc203638421)

[Supplementary Table S5. Trustworthiness assessment for trials without IPD 25](#_Toc203638422)

[Supplementary Table S6. Two-stage IPD meta-analysis for all outcomes 27](#_Toc203638423)

[Supplemental Table S7. Individual patient-level subgroup analyses (Treatment-covariate interaction) 28](#_Toc203638424)

[Supplemental Table S8. Study-level subgroup analyses 29](#_Toc203638425)

[Supplementary Figure S1a-b. Funnel plots for live birth and clinical pregnancy stratified by RCTs with and without IPD 31](#_Toc203638426)

# Supplementary Table S1. Search strategies

| Databases | Search strategy |
| --- | --- |
| **Cochrane Gynaecology and Fertility Group (CGF) Specialised Register**  **ProCite platform**  **Searched from inception to 23 November 2023*** (the last date of entry to the Register) | Keywords CONTAINS "IVF" or "in vitro fertilization" or "in‐vitro fertilisation" or "ICSI" or "intracytoplasmic sperm injection" or "ET" or "Embryo" or "in‐vitro fertilization" or "Embryo Transfer" or "Embryo Transfer‐uterine" or "blastocyst transfer" or Title CONTAINS "IVF" or "in vitro fertilization" or "in‐vitro fertilisation" or "ICSI" or "intracytoplasmic sperm injection" or "Embryo" or "in‐vitro fertilization" or "ET" or "Embryo" or "in‐vitro fertilization" or "Embryo Transfer" or "Embryo Transfer‐uterine" or "blastocyst transfer"  AND  Keywords CONTAINS "HCG " or "human chorionic gonadotrophin" or "human chorionic gonadotropin" or "recombinant HCG" or "rhCG" or Title CONTAINS "HCG " or "human chorionic gonadotrophin" or "human chorionic gonadotropin" or "recombinant HCG" or "rhCG"  AND  Keywords CONTAINS "intrauterine human chorionic gonadotrophin" or "intrauterine" or "Intrauterine injection" or "intrauterine instillation "or "uterine cavity injection" or "endometrial" or "Endometrium" or "uterine" or Title CONTAINS "intrauterine human chorionic gonadotrophin" or "intrauterine" or "Intrauterine injection" or "intrauterine instillation "or "uterine cavity injection" or "Endometrium" or "uterine" |
| **Cochrane Central Register of Controlled Trials via the**  **Cochrane Register of Studies Online**  **Web platform**  **Searched from inception to 27 January 2026** | #1 MESH DESCRIPTOR Reproductive Techniques, Assisted EXPLODE ALL TREES  #2 ( embryo* adj2 transfer*):TI,AB,KY  #3 (blastocyst* adj2 transfer*):TI,AB,KY  #4 (assisted reproduct* ):TI,AB,KY  #5 (ivf or icsi):TI,AB,KY  #6 (in vitro fertili?ation):TI,AB,KY  #7 (intracytoplasmic sperm injection*):TI,AB,KY  #8 #1 OR #2 OR #3 OR #4 OR #5 OR #6 OR #7  #9 MESH DESCRIPTOR Chorionic Gonadotropin EXPLODE ALL TREES  #10 (Human Chorionic Gonadotrop?in adj7 intrauter*):TI,AB,KY  #11 (Human Chorionic Gonadotrop?in adj7 uter*):TI,AB,KY  #12 ((endometri* adj2 infusion*) and chorionic):TI,AB,KY  #13 ((intra?uter* adj2 infusion*) and chorionic):TI,AB,KY  #14 ((intra?uter* adj2 instillation) and chorionic):TI,AB,KY  #15 ((endometri* adj2 injection*) and chorionic):TI,AB,KY  #16 ((intra?uter* adj2 injection*) and chorionic):TI,AB,KY  #17 ((intra?uter* adj2 administration) and chorionic):TI,AB,KY 41  #18 (intrauter* adj7 ?hcg):TI,AB,KY  #19 #9 OR #10 OR #11 OR #12 OR #13 OR #14 OR #15 OR #16 OR #17 OR #18  #20 #8 AND #19 |
| **MEDLINE**  **Ovid platform**  **Searched from 1946 to 27 January 2026** | 1 exp reproductive techniques, assisted/  2 assisted reproducti*.tw.  3 embryo transfer$.tw.  4 in vitro fertili?ation.tw.  5 assisted reproduct*.tw.  6 (ivf or icsi).tw.  7 infertil.tw.  8 intracytoplasmic sperm injection$.tw.  9 (blastocyst adj2 transfer$).tw.  10 or/1-9  11 exp Chorionic Gonadotropin/ad, tu, th [Administration & Dosage, Therapeutic Use, Therapy]  12 (Human Chorionic Gonadotrop?in adj7 intrauter$).tw.  13 (Human Chorionic Gonadotrop?in adj7 uter$).tw.  14 (Human Chorionic Gonadotrop?in adj7 intra-uter$).tw.  15 ((endometri$ adj2 infusion$) and chorionic).tw.  16 ((endometri$ adj2 ?instillation) and chorionic).tw.  17 ((intra?uter$ adj2 infusion$) and chorionic).tw.  18 ((intra?uter$ adj2 ?instillation) and chorionic).tw.  19 ((endometri$ adj2 injection$) and chorionic).tw.  20 ((intra?uter$ adj2 injection$) and chorionic).tw.  21 ((intra?uter$ adj2 administration) and chorionic).tw.  22 ((endometri$ adj2 administration) and chorionic).tw.  23 (intrauter$ adj7 ?hcg).tw.  24 (intra-uter$ adj7 ?hcg).tw.  25 (uter$ adj7 ?hcg).tw.  26 or/11-25  27 10 and 26 (  28 randomized controlled trial.pt.  29 controlled clinical trial.pt.  30 randomized.ab.  31 randomised.ab.  32 placebo.tw.  33 clinical trials as topic.sh.  34 randomly.ab.  35 trial.ti.  36 (crossover or cross-over or cross over).tw.  37 or/28-36  38 exp animals/ not humans.sh.  39 37 not 38  40 27 and 39 |
| **Embase**  **Ovid platform**  **Searched from 1980 to 27 January 2026** | 1 infertility therapy/  2 embryo$ transfer$.tw.  3 in vitro fertili?ation.tw.  4 assisted reproduct*.tw.  5 infertil*.tw.  6 intracytoplasmic sperm injection$.tw.  7 (blastocyst adj2 transfer$).tw.  8 (ivf or icsi).tw.  9 or/1-8  10 (Human Chorionic Gonadotrop?in adj7 intrauter$).tw.  11 (Human Chorionic Gonadotrop?in adj7 uter$).tw.  12 (intrauter$ adj7 ?hcg).tw.  13 chorionic gonadotropin/dt, ut [Drug Therapy, Intrauterine Drug Administration]  14 (uter$ adj3 ?hcg).tw.  15 ((endometri$ adj2 infusion$) and chorionic).tw.  16 ((endometri$ adj2 ?instillation) and chorionic).tw.  17 ((intra?uter$ adj2 infusion$) and chorionic).tw.  18 ((intra?uter$ adj2 ?instillation) and chorionic).tw.  19 ((endometri$ adj2 injection$) and chorionic).tw.  20 ((intra?uter$ adj2 injection$) and chorionic).tw.  21 ((intra?uter$ adj2 administration) and chorionic).tw.  22 ((endometri$ adj2 administration) and chorionic).tw.  23 or/10-22  24 9 and 23  25 Clinical Trial/  26 Randomized Controlled Trial/  27 exp randomization/  28 Single Blind Procedure/  29 Double Blind Procedure/  30 Crossover Procedure/  31 Placebo/  32 Randomi?ed controlled trial$.tw.  33 Rct.tw.  34 random allocation.tw.  35 randomly allocated.tw.  36 allocated randomly.tw.  37 (allocated adj2 random).tw.  38 Single blind$.tw.  39 Double blind$.tw.  40 ((treble or triple) adj blind$).tw.  41 placebo$.tw.  42 prospective study/  43 or/25-42  44 case study/  45 case report.tw.  46 abstract report/ or letter/  47 or/44-46  48 43 not 47  49 24 and 48 |
| **PsycINFO**  **Ovid platform**  **Searched from 1806 to 27 January 2026** | 1 exp reproductive technology/  2 in vitro fertili?ation.tw.  3 (icsi or ivf).tw.  4 intracytoplasmic sperm injection$.tw.  5 (blastocyst adj2 transfer$).tw.  6 (embryo$ adj2 transfer$).tw.  7 or/1-6  8 exp Gonadotropic Hormones/  9 Human Chorionic Gonadotrop?in*.tw.  10 HCG.tw.  11 or/8-10  12 7 and 11 |

Footnotes:

*****The Cochrane Gynaecology and Fertility Group (CGF) Specialised Register has not been updated since 23^rd^ November 2023.

# Supplementary Table S2. Characteristics of non-participated reports

| Report | Country | Participant size | Reason not to participate |
| --- | --- | --- | --- |
| ^1^Aaleyasin 2015 | Iran | 483 | Never received a response |
| ^2^Aly 2018 | UK | N/A | Author’s response: I’m afraid that I have no knowledge of this study or any involvement in it. |
| ^3^Bhat 2014 | India | 32 | Never received a response |
| ^4^Cambiaghi 2013 | Brazil | 44 | Never received a response |
| ^5^Dutta 2018 | India | 74 | Never received a response |
| ^6^Eskander 2016 | Saudi Arabia | 240 | Agreed to share data, but did not receive response afterwards |
| ^7^Firouzabadi 2016 | Iran | 159 | Never received a response |
| ^8^Hosseini 2016 | Iran | 100 | Never received a response |
| ^9^Hosseinisadat 2020 | Iran | 126 | Never received a response |
| ^10^Huang 2016 | China | 110 | The abstract and table of baseline characteristic were received, then author’s response: sorry, that’s all |
| ^11^Kokkali 2014 | Greece | 194 | Agreed to share data, but data in the wrong format was received |
| ^12^Leao 2013 | Brazil | 36 | Never received a response |
| ^13^Liu 2019 | China | 303 | Never received a response |
| ^14^Mansour 2011 | Egypt | 210 | Corresponding author deceased |
| ^15^Mostjaren 2017 | Iran | 100 | Never received a response |
| ^16^Navali 2016 | Iran | 158 | Never received a response |
| ^17^Rezaei 2018 | Iran | 80 | Never received a response |
| ^18^Santibanez 2014 | Mexico | 210 | Never received a response |
| ^19^Singh 2014 | India | 216 | Agreed to share data, but did not receive response afterwards |
| ^20^Wang 2019 | China | 140 | Never received a response |
| ^21^Zarei 2014 | Iran | 182 | Never received a response |

References:

1. Aaleyasin A, Aghahosseini M, Rashidi M, Safdarian L, Sarvi F, Najmi Z, Mobasseri A, Amoozgar B. In vitro fertilization outcome following embryo transfer with or without preinstillation of human chorionic gonadotropin into the uterine cavity: a randomized controlled trial. Gynecologic and Obstetric Investigation 2015;79: 201-205.

2. Aly J, Miller D. The effect of timing intrauterine human chorionic gonadotropin injection before embryonic transfer on intracytoplasmic sperm injection outcomes: a prospective randomized study. BJOG 2018;125: 65-66.

3. Bhat VV, Dutta I, Dutta DK, Gcitha MD. Outcome of Intrauterine Injection of Human Chorionic Gonadotropin before Embryo Transfer in Patients with Previous Ivf/Icsi Failure: A Randomized Study. J South Asian Feder Obst Gynae 2014;6(1):15-17.

4. Cambiaghi A, Leao R, Alvarez A, Nascimento P. Intrauterine injection of human chorionic gonadotropin before embryo transfer may improve clinical pregnancy and implantation rates in blastocysts transfers. Fertility and Sterility 2013;100: S121.

5. Dutta I. A randomized study showing the outcome of intrauterine injection of human chorionic gonadotropin before embryo transfer in patients with previous IVF/ICSI failure. Journal of obstetrics and gynaecology research 2017;43: 170‐171.

6. Eskandar MA, Al-Emain MA, Atwan YL, Bakar SD. Does Intrauterine Injection of Human Chorionic Gonadotropin Before Embryo Transfer Improve the Pregnancy Rate In Vitro Fertilization/Intracytoplasmic Sperm Injection (IVFICSI) Cycles? A Prospective Randomized Controlled Trial Reproductive Sciences. 2016; Vol. 23, pp. 102A-102A.

7. Firouzabadi RD, Janati S, Razi MH. The effect of intrauterine human chorionic gonadotropin injection before embryo transfer on the implantation and pregnancy rate in infertile patients: A randomized clinical trial. International Journal of Reproductive Biomedicine 2016;14: 657.

8. Hosseini RS, Farzadi L, Abdollahi S, Nouri M, Ghasemzadeh A, Hamdi K, Soleimanpour H. Effect of intrauterine injection of human chorionic gonadotropin before frozen-thawed embryo transfer on implantation and clinical pregnancy rate: a randomized controlled trial. International Journal of Women’s Health and Reproduction Sciences 2016;4: 189-193.

9. Hosseinisadat R, Saeed L, Ashourzadeh S, Heidari SS, Habibzadeh V. Effects of human chorionic gonadotropin intrauterine injection on oocyte retrieval day on assisted reproductive techniques outcomes: An RCT. Int J Reprod Biomed 2021;19: 773-780.

10. Huang P, Wei L, Li X. A study of intrauterine infusion of human chorionic gonadotropin (hCG) before frozen-thawed embryo transfer after two or more implantation failures. Gynecological Endocrinology 2017;33: 67-69.

11. Kokkali G, Chronopoulou M, Baxevani E, Biba M, Angeli I, Fakiridou M, Katouna A, Markomichali C, Petroutsou K, Vaxevanoglou T. A randomised control pilot study of the use of intrauterine human chorionic gonadotropin injection before embryo transfer in egg recipient cycles. Human Reproduction 2014;29: i208.

12. Leao R, Cambiaghi A, Leao B, Alvarez P, Figueiredo P. Intrauterine injection of human chorionic gonadotropin before embryo transfer may improve the pregnancy rates in in vitro fertilization cycles of patients with repeated implantation failures Proceedings of the 5th IVI International Congress, Seville, Spain. 2013.

13. Liu X, Ma D, Wang W, Qu Q, Zhang N, Wang X, Fang J, Ma Z, Hao C. Intrauterine administration of human chorionic gonadotropin improves the live birth rates of patients with repeated implantation failure in frozen-thawed blastocyst transfer cycles by increasing the percentage of peripheral regulatory T cells. Archives of Gynecology and Obstetrics 2019;299: 1165-1172.

14. Mansour R, Tawab N, Kamal O, El-Faissal Y, Serour A, Aboulghar M, Serour G. Intrauterine injection of human chorionic gonadotropin before embryo transfer significantly improves the implantation and pregnancy rates in in vitro fertilization/intracytoplasmic sperm injection: a prospective randomized study. Fertil Steril 2011;96: 1370-1374.e1371.

15. Mostajeran F, Godazandeh F, Ahmadi SM, Movahedi M, Jabalamelian SA. Effect of intrauterine injection of human chorionic gonadotropin before embryo transfer on pregnancy rate: A prospective randomized study. Journal of Research in Medical Sciences: The Official Journal of Isfahan University of Medical Sciences 2017;22.

16. Navali N, Gassemzadeh A, Farzadi L, Abdollahi S, Nouri M, Hamdi K, Mallah F, Jalilvand F. Intrauterine administration of hCG immediately after oocyte retrieval and the outcome of ICSI: a randomized controlled trial. Human Reproduction 2016;31: 2520-2526.

17. Rezaei M, Frhadifar F, Moradi G, Khalatbari I. Evaluation of the impact of 1,000-unit intrauterine injection of human chorionic gonadotropin prior to in vitro fertilization on success rate of implantation in infertile women: A double-blind randomized trial. Journal of South Asian Federation of Obstetrics and Gynaecology 2018;10: 10-14.

18. Santibañez A, García J, Pashkova O, Colín O, Castellanos G, Sánchez AP, De la Jara JF. Effect of intrauterine injection of human chorionic gonadotropin before embryo transfer on clinical pregnancy rates from in vitro fertilisation cycles: a prospective study. Reprod Biol Endocrinol 2014;12: 9.

19. Singh R, Singh M. Intra-uterine administration of human chorionic gonadotrophin (hCG) before embryo transfer in recurrent implantation failure (RIF) patients improves implantation and pregnancy rates in IVF-ICSI cycles Human Reproduction. Oxford Univ Press 2014;29: 79-79.

20. Wang M, Deng H, Ye H. [Intrauterine injection of human chorionic gonadotropin improves pregnancy outcome in patients with repeated implantation failure in frozen-thawed embryo transfer]. Zhong Nan Da Xue Xue Bao Yi Xue Ban 2019;44: 1247-1251.

21. Zarei A, Parsanezhad ME, Younesi M, Alborzi S, Zolghadri J, Samsami A, Amooee S, Aramesh S. Intrauterine administration of recombinant human chorionic gonadotropin before embryo transfer on outcome of in vitro fertilization/intracytoplasmic sperm injection: A randomized clinical trial. Iranian journal of reproductive medicine 2014;12: 1.

# Supplementary Table S3. Characteristics of excluded reports and ongoing trials

| Reports | Reason for exclusion |
| --- | --- |
| ^1^Aaleyasin 2015 | Secondary reports |
| ^2^Badehnoosh 2014 | Secondary reports |
| ^3^Boonsuk 2015 | Secondary reports |
| ^4^Bienert 2021 | Irrelevant outcome of interest |
| ^5^Ghasemzadeh 2022 | Irrelevant intervention of interest |
| ^6^Hafezi 2018 | Secondary reports |
| ^7^Hosseini 2016 | Secondary reports |
| ^8^Hong 2014 | Secondary reports |
| ^9^Jahanshahi 2022 | Irrelevant design of interest |
| ^10^Mansour 2011 | Secondary reports |
| ^11^Mustapha 2022 | Irrelevant population of interest |
| ^12^Naghshineh 2022 | Irrelevant design of interest |
| ^13^Navali 2016 | Secondary reports |
| ^14^Strug 2016 | Irrelevant population of interest |
| ^15^Torky 2022 | Retracted trial |
| ^16^Wadhwa 2021 | Irrelevant population of interest |
| ^17^Zarei 2014 | Secondary reports |
| Ongoing trials |  |
| ^18^Gibreel 2014 | Aborted trial identified in the trial registration |
| ^19^Firouzabadi 2020 | Ongoing trial |
| ^20^Gedela 2022 | Ongoing trial |
| ^21^Fakih 2021 | Irrelevant population of interest |
| ^22^Gooya 2015 | Ongoing trial |
| ^23^Hosseinimousa | Ongoing trial |
| ^24^Hradecký 2015 | Ongoing trial |
| ^25^Ido 2014 | Ongoing trial |
| ^26^Jalali 2014 | Ongoing trial |
| ^27^Karimi 2020 | Ongoing trial |
| ^28^Khadem 2020 | Ongoing trial |
| ^29^Tarafdari 2025 | Ongoing trial |
| ^30^Xiangya 2016 | Ongoing trial |
| ^31^Ziaee 2017 | Ongoing trial |

References:

1. https://trialsearch.who.int/Trial2.aspx?TrialID=IRCT201208058469N2.

2. Badehnoosh B, Mohammadzadeh A, Sadeghi M, Akhondi M, Kazemnejad S, Sadaei-Jahromi N, Arjmand-Teimoori F, Zafardoost S, Fatemi F, Mokhtar S. The effects of intrauterine injection of human chorionic gonadotropin (hCG) before embryo transfer on the implantation rate in the intracytoplasmic sperm injection (ICSI) program. International Journal of Reproductive BioMedicine 2014;12: 10.

3. Laokirkkiat P, Thanaboonyawat I, Boonsuk S, Petyim S, Prechapanich J, Choavaratana R. Increased implantation rate after intrauterine infusion of a small volume of human chorionic gonadotropin at the time of embryo transfer: a randomized, double-blind controlled study. Arch Gynecol Obstet 2019;299: 267-275.

4. Bienert M, Habib P, Buck V, Classen-Linke I, Skoblo R, Rösing B. Intrauterine hCG application increases expression of endothelial cell-cell adhesion molecules in human. Arch Gynecol Obstet 2021;304: 1587-1597.

5. Akbari Asbagh, F., Ghasemzadeh, F., Ebrahimi, M., Davari-Tanha, F., Feizabad, E., Akbari Asbagh, P., & Hosseini Quchani, S. (2023). Effect of intramuscular injection of human chorionic gonadotropin on endometrium preparation in frozen-thawed embryo transfer cycle: A randomized clinical trial. Caspian journal of internal medicine, 14(2), 185–191. https://doi.org/10.22088/cjim.14.2.185

6. Hafezi M, Madani T, Arabipoor A, Zolfaghari Z, Sadeghi M, Ramezanali F. The effect of intrauterine human chorionic gonadotropin flushing on live birth rate after vitrified-warmed embryo transfer in programmed cycles: a randomized clinical trial. Arch Gynecol Obstet 2018;297: 1571-1576.

7. https://trialsearch.who.int/Trial2.aspx?TrialID=IRCT2014110612146N5.

8. Hong KH, Forman EJ, Werner MD, Upham KM, Gumeny CL, Winslow AD, Kim TJ, Scott RT, Jr. Endometrial infusion of human chorionic gonadotropin at the time of blastocyst embryo transfer does not impact clinical outcomes: a randomized, double-blind, placebo-controlled trial. Fertil Steril 2014;102: 1591-1595.e1592.

9. Jahanshahi M, Aleyasin A, Aghahosseini M, Najafian A, Nashtaei MS, Hosseinimousa S. The effect of intrauterine hCG injection before embryo transfer on pregnancy rate in frozen embryo transfer cycles. Annals of Medicine and Surgery 2022;79: 104091.

10. https://trialsearch.who.int/Trial2.aspx?TrialID=NCT01030393.

11. Mustapha H, Lahimer M, Makni M, Bannour I, Kaabia O, Derouich M, Ferjaoui MA, Arfaoui R, Zaouali M, Ajina M. Effect of intrauterine administration of human chorionic gonadotropin one day before fresh blastocyst transfer on clinical outcomes: a quasi-experimental study. Pan Afr Med J 2022;42: 27.

12. Naghshineh E, Dehghani Mohammadabadi R, Mehrabian F, Ghasemi Tehrani H, Tarrahi MJ. Intrauterine Instillation of Human Chorionic Gonadotropin with Intrauterine Insemination Catheter Around the Golden Time of Embryo Transfer Does Not Improve In Vitro Fertilization /Intracytoplasmic Sperm Injection Outcomes in Infertile Women: A Randomized Controlled Trial. Rep Biochem Mol Biol 2022;11: 358-366.

13. https://trialsearch.who.int/Trial2.aspx?TrialID=IRCT201206165485N4.

14. Strug MR, Su R, Young JE, Dodds WG, Shavell VI, Díaz-Gimeno P, Ruíz-Alonso M, Simón C, Lessey BA, Leach RE et al. Intrauterine human chorionic gonadotropin infusion in oocyte donors promotes endometrial synchrony and induction of early decidual markers for stromal survival: a randomized clinical trial. Hum Reprod 2016;31: 1552-1561.

15. Torky H, El-Desouky E-S, El-Baz A, Aly R, El-Taher O, Shata A, Hussein A, Marie H, Deif O, Eldemery A. Effect of intra uterine granulocyte colony stimulating factor vs. human chorionic gonadotropin at ovum pick up day on pregnancy rate in IVF/ICSI cases with recurrent implantation failure. JBRA Assisted Reproduction 2022;26: 274.

16. Wadhwa L, Rani A. Impact of Intrauterine Administration of Human Chorionic Gonadotropin before Intrauterine Insemination in Infertile Women: A Randomized Controlled Trial. J Hum Reprod Sci 2021;14: 156-161.

17. https://trialsearch.who.int/Trial2.aspx?TrialID=IRCT2012121711790N1.

References for aborted trial:

18. https://trialsearch.who.int/Trial2.aspx?TrialID=NCT02329197

References for ongoing trials:

19. https://trialsearch.who.int/Trial2.aspx?TrialID=IRCT20200305046702N1

20. Gedela D, Mantravadi K, Sundari S. O-023 A randomized control trial on the effect of intrauterine HCG flushing on reproductive outcomes. Human Reproduction 2022;37: deac104. 023.

21. https://trialsearch.who.int/Trial2.aspx?TrialID=LBCTR2021034751

22. https://trialsearch.who.int/Trial2.aspx?TrialID=IRCT2015020921008N1

23. https://trialsearch.who.int/Trial2.aspx?TrialID=IRCT20230803059016N1

24. https://trialsearch.who.int/Trial2.aspx?TrialID=DRKS00026832

25. https://trialsearch.who.int/Trial2.aspx?TrialID=NCT01933126

26. https://trialsearch.who.int/Trial2.aspx?TrialID=IRCT2013110815320N1

27. https://trialsearch.who.int/Trial2.aspx?TrialID=IRCT20200213046475N1

28. https://trialsearch.who.int/Trial2.aspx?TrialID=IRCT20181030041503N2

29. https://trialsearch.who.int/Trial2.aspx?TrialID=IRCT20250201064590N1

30. https://trialsearch.who.int/Trial2.aspx?TrialID=NCT03682614

31. https://trialsearch.who.int/Trial2.aspx?TrialID=IRCT2017041733486N1

# Supplementary Table S4. Trustworthiness assessment for trials with IPD

| Integrity domain and items | Abdallah 2021 | Hafezi 2018 | Hong 2014 | Laokirkkiat 2019 | Matsumoto 2015 | Wirleitner 2015a | Wirleitner 2015b | Badehnoosh 2014^17^ |
| --- | --- | --- | --- | --- | --- | --- | --- | --- |
| Aggregate data/publication-level checks |  |  |  |  |  |  |  |  |
| 1. Retraction notices and expression of concern |  |  |  |  |  |  |  |  |
| 1.1 Retraction notice- study of interest | No issues | No issues | No issues | No issues | No issues | No issues | No issues | No issues |
| 1.2 Retraction notice(s)- other study/ies by same authors | No issues | No issues | No issues | No issues | No issues | No issues | No issues | Many/major issues |
| 1.3 Expression of concern (EOC)- study of interest | No issues | No issues | No issues | No issues | No issues | No issues | No issues | No issues |
| 1.4 Expression of concern- other study/ies by same authors | No issues | No issues | No issues | No issues | No issues | No issues | No issues | Many/major issues |
| 2. Provision of Individual participant data (IPD) |  |  |  |  |  |  |  |  |
| 2.1 IPD not available or not provided on request | No issues | No issues | No issues | No issues | No issues | No issues | No issues | No issues |
| 3. Communication |  |  |  |  |  |  |  |  |
| 3.1 Lack of trialist engagement in communication | No issues | No issues | No issues | No issues | No issues | No issues | No issues | No issues |
| 4. Ethics approval |  |  |  |  |  |  |  |  |
| 4.1 Absent or inadequate ethics approval | No issues | No issues | No issues | No issues | Some/minor issues^9^ | No issues | No issues | Some/minor issues |
| 5. Trial registration / protocol |  |  |  |  |  |  |  |  |
| 5.1 Absent or retrospective trial registration +/- publicly available protocol | No issues | No issues | No issues | No issues | Some/minor issues^10^ | Some/minor issues^12^ | Some/minor issues^15^ | Some/minor issues |
| 6. Randomisation |  |  |  |  |  |  |  |  |
| 6.1 Randomisation - baseline balance/imbalance across groups | No issues | No issues | No issues | No issues | No issues | No issues | No issues | Some/minor issues |
| 7. Plausibility |  |  |  |  |  |  |  |  |
| 7.1 Implausible recruitment rate | No issues | No issues | No issues | No issues | Some/minor issues^11^ | No issues | No issues | No issues |
| 7.2 Implausible follow-up | No issues | No issues | No issues | No issues | No issues | No issues | No issues | No issues |
| 7.3 Implausible results | No issues | No issues | No issues | No issues | No issues | No issues | No issues | No issues |
| 7.4 Implausible author group | No issues | No issues | No issues | No issues | No issues | No issues | No issues | No issues |
| Overall Judgement- aggregate data/publication-level checks | No concerns | No concerns | No concerns | No concerns | Some concerns | No concerns | No concerns | Major concerns |
|  |  |  |  |  |  |  |  |  |
| Individual participant data checks |  |  |  |  |  |  |  |  |
| 1. Unusual or repeated data patterns |  |  |  |  |  |  |  |  |
| 1.1 Repeating data patterns within baseline variables | No issues | No issues | No issues | No issues | No issues | No issues | No issues | N/A |
| 1.2 Repeating data patterns across baseline variables | No issues | No issues^3^ | No issues | No issues | No issues | Some/minor issues^13^ | No issues | N/A |
| 1.3 Repeating data patterns across baseline variables and rare variables | No issues | No issues | No issues | No issues | No issues | No issues | No issues | N/A |
| 1.4 Bias in the terminal (rightmost) digits | Some/minor issues^1^ | Some/minor issues^4^ | Not applicable | No issues | No issues | No issues | No issues | N/A |
| 2. Baseline characteristics |  |  |  |  |  |  |  |  |
| 2.1 Excessively homogeneous distribution of binary baseline variables, i.e. loss of independence or serial correlation across consecutive observations | No issues | No issues | No issues | No issues | Not applicable | No issues | No issues | N/A |
| 2.2 Excessive imbalances between groups in continuous baseline variables | No issues | No issues | No issues | No issues | No issues | No issues | No issues | N/A |
| 2.3 Excessive imbalances in baseline categorical variables between groups | No issues | No issues | No issues | No issues | Not applicable | No issues | No issues | N/A |
| 2.4 Significant difference in variance of continuous baseline variables between groups | No issues | No issues | No issues | No issues | No issues | No issues | No issues | N/A |
| 3. Correlations |  |  |  |  |  |  |  |  |
| 3.1 No association between variables known to be highly correlated | No issues | Some/minor issues^5^ | Not applicable | Not applicable | Not applicable | Not applicable | Not applicable | N/A |
| 4. Date violations |  |  |  |  |  |  |  |  |
| 4.1 Individual enrolment dates do not fit within study start and end dates | Not applicable | No issues^6^ | Not applicable | Not applicable | Not applicable | Not applicable | Not applicable | N/A |
| 4.2 Dates (or visits) are not in logical order | Not applicable | No issues | Not applicable | Not applicable | Not applicable | Not applicable | Not applicable | N/A |
| 5. Patterns of allocation |  |  |  |  |  |  |  |  |
| 5.1 Non-random allocation patterns - plot | Not applicable | No issues | Not applicable | Not applicable | Not applicable | Not applicable | Not applicable | N/A |
| 5.2 Non-random allocation patterns - statistical test | Not applicable | No issues^7^ | Not applicable | Not applicable | Not applicable | Not applicable | Not applicable | N/A |
| 5.3 Unexpected imbalance in randomisation day of week | Not applicable | No issues | Not applicable | Not applicable | Not applicable | Not applicable | Not applicable | N/A |
| 6. Internal inconsistencies |  |  |  |  |  |  |  |  |
| 6.1 Inconsistent or illogical values across variables within individual participants | No issues | No issues | No issues | No issues | No issues | Not applicable | No issues | N/A |
| 7. External inconsistencies |  |  |  |  |  |  |  |  |
| 7.1 IPD do not correspond to publications or reports | Some/minor issues^2^ | No issues | No issues | Some/minor issues^8^ | Not applicable | Some/minor issues^14^ | Some/minor issues^16^ | N/A |
| 8. Plausibility of data |  |  |  |  |  |  |  |  |
| 8.1 Too few missing data or missing data are overly similar between groups | No issues | No issues | No issues | No issues | No issues | No issues | No issues | N/A |
| 8.2 Implausible event rates - outcomes & demographics | No issues | No issues | No issues | No issues | No issues | No issues | No issues | N/A |
| Overall Judgement- individual participant data checks | No concerns | Some concerns | No concerns | No concerns | No concerns | No concerns | No concerns | N/A |

Footnotes:

1. BMI and fsh peaking at 0- probably rounding or measurement issue

2. Duration of infertility slightly different, small clashed in other variables e.g. precyc (probably non consequential difference of 1)

3. Discrepancies but likely the consequence of merging two control groups

4. Big peak of ages with terminal digit of 0, can’t think of a rounding reason for this- seemingly this is driven by 20 people who are 40 years old

5. No correlation between fsh and age

6. Slight discrepancies, likely due to the use of two different calendar systems

7. Initial discrepancies, however these were the consequence of merging two control groups

8. Small difference in means of age and BMI

9. Not reported however only abstract- maybe we need to query

10. Not reported however only abstract- maybe we need to query

11. Only two authors and they recruited 173 patients, however it doesn’t say time scale so it’s hard to evaluate (abstract only)

12. Previously reported in an abstract, not reported in this report

13. Many duplicates however only two continuous variables so this is plausible

14. Age and BMI slightly differ to publication

15. Previously reported in an abstract, not reported in this report

16. BMI and age slightly differ from the publication

17. Due to integrity concerns related to retractions from other publications from the same author, we have decided to exclude this trial. For the present trial there is only an abstract published which is insufficient information to evaluate integrity given a few items have already been rated as having concerns.

# Supplementary Table S5. Trustworthiness assessment for trials without IPD

| Study Name | Governance | Author Group | Plausibility of Intervention Usage | Timeframe | Drop-Out Rates | Baseline Characteristics | Outcomes |
| --- | --- | --- | --- | --- | --- | --- | --- |
| Aaleyasin 2015 | Major concerns^1^ | No concerns | No concerns | No concerns | No concerns | No concerns | No concerns |
| Bhat 2014 | Major concerns^2^ | No concerns | Major concerns^3^ | No concerns | No concerns | Some concerns^4^ | Major concerns^5^ |
| Firouzabadi 2016 | Major concerns^6^ | Some concerns^7^ | Major concerns^3^ | No concerns | No concerns | No concerns | No concerns |
| Hosseini 2016 | Major concerns^6^ | Major concerns^8^ | Major concerns^3^ | No concerns | No concerns | Some concerns^4^ | Some concerns^9^ |
| Hosseinisadat 2020 | No concerns | No concerns | Major concerns^3^ | No concerns | Some concerns^10^ | No concerns | Major concerns^11^ |
| Huang 2016 | Major concerns^12^ | Some concerns^7^ | Major concerns^3^ | No concerns | No concerns | Major concerns^13^ | Some concerns^14^ |
| Liu 2019 | Major concerns^12^ | Major concerns^8^ | Major concerns^15^ | No concerns | No concerns | No concerns | No concerns |
| Mansour 2011 | Major concerns^16^ | No concerns | Major concerns^17^ | No concerns | Some concerns^18^ | No concerns | No concerns |
| Mostajeran 2017 | Major concerns^12^ | No concerns | Major concerns^3^ | No concerns | No concerns | No concerns | No concerns |
| Navali 2016 | Major concerns^19^ | Major concerns^8^ | No concerns | Major concerns^20^ | Some concerns^18^ | No concerns | Some concerns^21^ |
| Rezaei 2018 | Major concerns^22^ | Some concerns^23^ | Major concerns^3^ | No concerns | No concerns | No concerns | No concerns |
| Santibanez 2014 | Major concerns^12^ | No concerns | Major concerns^15^ | No concerns | No concerns | Some concerns^24^ | No concerns |
| Wang 2019 | Major concerns^12^ | Some concerns^7^ | Major concerns^3^ | No concerns | No concerns | No concerns | No concerns |
| Zarei 2014 | Major concerns^25^ | Major concerns^8^ | No concerns | No concerns | Some concerns^18^ | Some concerns^4^ | No concerns |

Footnotes:

1. No trial registration. Recruited >15% than intended (483 recruited vs 410 planned).

2. No trial registration, no information on intended sample size and absent description of research ethics.

3. No description of allocation concealment.

4. Less than five baseline characteristics presented; some important prognostic factors not reported.

5. Publication did not report implantation/pregnancy rates, despite concluding that the intervention "statistically improved the implantation and pregnancy rates".

6. Retrospective trial registration. Retrospective registration defined as any date after recruitment of first participant.

7. Less than four authors on publication.

8. At least one author with earlier retracted studies.

9. No effect sizes were reported, however calculated OR (3.75) for clinical pregnancy is higher than the OR in MAs (OR 2.02).

10. Identically sized groups for analysis after excluding participants who did not receive allocated treatment (no rationale or reason provided).

11. The primary outcome in registration (chemical pregnancy) was reported as a secondary outcome in publication. The five primary outcomes in publication were not pre-specified in registration (no rationale/reason provided), and these are not outcomes as they occur before randomisation.

12. Absent trial registration.

13. Mean age and BMI are exactly the same between placebo and control groups. Standard errors are exactly the same too.

14. No effect sizes were reported, however calculated OR ( 3.15 for control group) for clinical pregnancy is much higher than MAs (OR 2.02). The calculated OR for placebo group was 1.31 - comparable to MA.

15. Authors refer to study as a prospective cohort/observational study despite using computer randomisation for treatment allocation; no description of allocation concealment.

16. Estimated enrolment was initially 900 (according to 10/12/2009 study record), but 472 were actually enrolled (according to 18/08/2012 study record). The planned sample size was 387 in the paper. Recruited > 15% than intended.

17. Randomised using sealed dark envelopes into two groups, but method of randomisation sequence generation not clear. Conducted two interim analyses which were not pre-specified in the registration. No description of allocation concealment.

18. Patients lost to follow-up with insufficient rationale provided.

19. Target sample size was 64 in the registration, but 160 according to the sample size calculation in the paper and 158 were randomised (discrepancy >15%).

20. Recruitment ended in Feb 2016, and then submitted in March and published in August 2016 (short timeframe given that they measured abortion rate before 20 weeks after ET).

21. Large effect size for clinical pregnancy (reported OR=3.08 but should be 5.25); larger than MA with an OR of 2.02.

22. Absent trial registration. Published in 2018 but no time period for study reported.

23. First author published at least one RCT per year.

24. Error in average age of control group (7.3 years old).

25. Retrospective registration. Notes ethics was obtained and approval number in registration. Ethics approval date was after recruitment start.

# Supplementary Table S6. Two-stage IPD meta-analysis for all outcomes

| Outcomes | Number of RCTs | Number of participants | Odds ratio | 95%CI | Tau2 | I2 |
| --- | --- | --- | --- | --- | --- | --- |
| Live birth | 7 | 2244 | 1.01 | 0.82-1.23 | 0.0059 | 0% |
| Clinical pregnancy | 7 | 2244 | 1.05 | 0.81-1.37 | 0.0291 | 28.3% |
| Ongoing pregnancy^#^ | 7 | 2244 | 1.03 | 0.83-1.27 | 0.0092 | 0% |
| Multiple pregnancy* | 5 | 1865 | 1.50 | 0.55-4.14 | 0.2863 | 36.4% |
| Miscarriage | 7 | 2244 | 1.22 | 0.89-1.67 | 0.0149 | 0% |
| Ectopic pregnancy^ | / | / | / | / | / | / |

Footnotes:

# Live birth was used as a surrogate for ongoing pregnancy for Hafezi 2018, Laokirkkiat 2019, and Wirleitner 2015a, b

* Two studies were not included in the two-stage model due to the presentence of 0 event in at least one group (Matsumoto 2015 & Wirleitner 2015a, b)

^ Two-stage model was not possible due to multiple studies with 0 event in at least one group (Abdallah 2021, Hafezi 2018, Matsumoto 2015), leaving only one study left in the meta-analysis.

# Supplemental Table S7. Individual patient-level subgroup analyses (Treatment-covariate interaction)

| Covariate | Outcome | Number of RCTs | Number of participants | Interaction odds ratio | Interaction 95% confidence intervals |
| --- | --- | --- | --- | --- | --- |
| Frozen embryo transfer (vs fresh embryo transfer) | Live birth | 2 | 500 | 0.78 | 0.13-4.59 |
|  | Clinical pregnancy | 3 | 681 | 0.81 | 0.33-1.94 |
| Blastocyst transfer (vs cleavage-stage embryo transfer) | Live birth* | / | / | / | / |
|  | Clinical pregnancy | 2 |  | 0.81 | 0.05-12.17 |

Footnote: * Two studies with participants undergoing fresh and frozen transfers included, but one study had 0 event in a strata. Meta-analysis was not performed with only one study left.

# Supplemental Table S8. Study-level subgroup analyses

| Subgroup | | Outcome | Number of RCTs | Number of participants | Odds ratio | 95% confidence intervals | P value for interaction |
| --- | --- | --- | --- | --- | --- | --- | --- |
| Type of control | Placebo | Live birth | 7 | 1344 | 1.06 | 0.81-1.38 | 0.088 |
|  | No treatment | Live birth | 1 | 90 | 0.43 | 0.16-1.15 |  |
|  | Placebo | Clinical pregnancy | 7 | 1344 | 1.09 | 0.80-1.39 | 0.276 |
|  | No treatment | Clinical pregnancy | 1 | 90 | 0.60 | 0.24-1.53 |  |
| Dosage of hCG | 1000 IU | Live birth | 1 | 197 | 1.06 | 0.81-2.12 | 0.685 |
|  | 500 IU | Live birth | 7 | 1147 | 1.00 | 0.76-1.32 |  |
|  | 1000 IU | Clinical pregnancy | 1 | 197 | 0.93 | 0.53-1.65 | 0.808 |
|  | 500 IU | Clinical pregnancy | 7 | 1147 | 1.08 | 0.77-1.52 |  |
| Fresh/frozen embryo transfer | Fresh embryo transfer | Live birth | 5 | 1547 | 0.96 | 0.70-1.31 | 0.482 |
|  | Frozen embryo transfer | Live birth | 5 | 697 | 1.10 | 0.68-1.77 |  |
|  | Fresh embryo transfer | Clinical pregnancy | 5 | 1547 | 1.10 | 0.69-1.75 | 0.570 |
|  | Frozen embryo transfer | Clinical pregnancy | 5 | 697 | 1.08 | 0.69-1.68 |  |
| Stage of embryo transfer | Cleavage-stage embryo transfer | Live birth | 3 | 420 | 1.03 | 0.27-3.97 | 0.953 |
|  | Blastocyst transfer | Live birth | 6 | 1824 | 1.00 | 0.76-1.33 |  |
|  | Cleavage-stage embryo transfer | Clinical pregnancy | 3 | 420 | 1.25 | 0.42-3.71 | 0.296 |
|  | Blastocyst transfer | Clinical pregnancy | 6 | 1824 | 0.99 | 0.73-1.35 |  |

# Supplementary Figure S1a-b. Funnel plots for live birth and clinical pregnancy stratified by RCTs with and without IPD

1a. Live birth

1b. Clinical pregnancy
